# Incidence dynamics and investigation of key interventions in a dengue outbreak in Ningbo City, China

**DOI:** 10.1371/journal.pntd.0007659

**Published:** 2019-08-15

**Authors:** Bo Yi, Yi Chen, Xiao Ma, Jia Rui, Jing-An Cui, Haibin Wang, Jia Li, Soi-Fan Chan, Rong Wang, Keqin Ding, Lei Xie, Dongliang Zhang, Shuli Jiao, Xuying Lao, Yi-Chen Chiang, Yanhua Su, Benhua Zhao, Guozhang Xu, Tianmu Chen

**Affiliations:** 1 Ningbo Municipal Center for Disease Control and Prevention, Ningbo City, Zhejiang Province, People’s Republic of China; 2 State Key Laboratory of Molecular Vaccinology and Molecular Diagnostics, School of Public Health, Xiamen University, Xiamen City, Fujian Province, People’s Republic of China; 3 School of Science, Beijing University of Civil Engineering and Architecture, Beijing, People's Republic of China; 4 Haishu District Center for Disease Control and Prevention, Ningbo City, Zhejiang Province, People’s Republic of China; 5 Center for Disease Control and Prevention, Health Bureau, Macao SAR, People’s Republic of China; Institute for Disease Modeling, UNITED STATES

## Abstract

**Background:**

The reported incidence of dengue fever increased dramatically in recent years in China. This study aimed to investigate and to assess the effectiveness of intervention implemented in a dengue outbreak in Ningbo City, Zhejiang Province, China.

**Methods:**

Data of a dengue outbreak were collected in Ningbo City in China by a field epidemiological survey according to a strict protocol and case definition. Serum specimens of all cases were collected for diagnosis and the virological characteristics were detected by using polymerase chain reaction (PCR) and gene sequencing. Vector surveillance was implemented during the outbreak to collect the larva and adult mosquito densities to calculate the Breteau Index (BI) and human biting rate (HBR), respectively. Data of monthly BI and light-trap density in 2018 were built to calculate the seasonality of the vector. A transmission mathematical model was developed to dynamic the incidence of the disease. The parameters of the model were estimated by the data of the outbreak and vector surveillance data in 2018. The effectiveness of the interventions implemented during the outbreak was assessed by the data and the modelling.

**Results:**

From 11 August to 8 September, 2018, a dengue outbreak was reported with 27 confirmed cases in a population of 5536-people community (community A) of Ningbo City. Whole E gene sequences were obtained from 24 cases and were confirmed as dengue virus type 1 (DENV-1). The transmission source of the outbreak was origin from community B where a dengue case having the same E gene sequence was onset on 30 July. *Aedes albopictus* was the only vector species in the area. The value of BI and HBR was 57.5 and 12 per person per hour respectively on 18 August, 2018 and decreased dramatically after interventions. The transmission model fitted well (*χ*^2^ = 6.324, *P* = 0.388) with the reported cases data. With no intervention, the total simulated number of the cases would be 1728 with a total attack rate (TAR) of 31.21% (95%CI: 29.99%– 32.43%). Case isolation and larva control (LC) have almost the same TAR and duration of outbreak (DO) as no intervention. Different levels of reducing HBR (rHBR) had different effectiveness with TARs ranging from 1.05% to 31.21% and DOs ranging from 27 days to 102 days. Adult vector control (AVC) had a very low TAR and DO. “LC+AVC” had a similar TAR and DO as that of AVC. “rHBR_100%_+LC”, “rHBR_100%_+AVC”, “rHBR_100%_+LC+AVC” and “rHBR_100%_+LC+AVC+Iso” had the same effectiveness.

**Conclusions:**

Without intervention, DENV-1 could be transmitted rapidly within a short period of time and leads to high attack rate in community in China. AVC or rHBR should be recommended as primary interventions to control rapid transmission of the dengue virus at the early stage of an outbreak.

## Author summary

Dengue has led to heavy disease burden in China. The reported incidence of the disease increased dramatically in recent years and cases have expanded from southern to central and northern part of China. In this study, the findings include that DENV-1 can transmit rapidly with a short period of time and leads to high attack rate in community, and that rHBR or AVC should be recommended as primary interventions to control rapid transmission of dengue virus at the early stage of an outbreak. Therefore, dengue outbreak is at high risk in many areas in China because of the potential high receptivity (widely distribution of *Ae*. *albopictus*) and vulnerability (high frequency of the importation) of the transmission. The high transmissibility of the virus makes it hard and urgent to control the outbreak. Delayed intervention (larvae control or case isolation) is hard to show its effectiveness and the interventions without delay are strongly recommended. Bed net or mosquito repellents were encouraged to use in the community to reduce HBR, and space spraying of insecticides were recommended to control adult vector during the outbreak.

## Introduction

Dengue Fever is a mosquito-borne infectious disease which is caused by 4 distinct serotypes of dengue virus (DENV-1, DENV-2, DENV-3, and DENV-4) [1, 2]. The disease is transmitted by female *Aedes* mosquitoes and has led to heavy disease burden in many countries [3, 4]. One recent research indicates 390 million dengue infections per year (95% credible interval 284–528 million) worldwide [3]. Another study estimates that 3.9 billion people in 128 countries are at risk of dengue infection [5]. The virus has become a leading cause of illness and death in tropics and subtropics. More important issues show that the virus has spread much wilder in recent decades. As reported by World Health Organization (WHO), only 9 countries had experienced severe dengue epidemics before 1970. But now, the disease is endemic in more than 100 countries in the WHO regions [1].

Dengue fever has been absent in China for about 30 years [2, 6, 7]. The reported incidence of the disease increased dramatically in recent years and cases have expanded from the coastal provinces of southern China and provinces adjacent to Southeast Asia to the central (Henan Province) and northern part (Shandong Province) of China [2]. The climate of most areas lying in tropical and subtropical zone is suitable for the reproduction of the vector and therefore provides a suitable environment for the disease transmission. When the virus imports in a desirable season, the transmission risk would be high. The rapid expansion of virus is an alert to conduct a risk assessment so as to understand the virus characteristics clearly. It could be better to investigate the transmission chain from an imported case to secondary cases in a non-epidemic area where there was no dengue transmission before than in epidemic area. Therefore, to understand a dengue outbreak in non-epidemic area has become an essential public health issue for the disease control and prevention.

From 2005 to 2014, no indigenous case was recorded in Ningbo City, Zhejiang Province, China. However, four indigenous cases occurred in the city in 2015. Then on 18 August, 2018, two suspected dengue cases were reported in two hospitals in Ningbo City. Serum specimens were collected and tested by polymerase chain reaction (PCR) method, two cases were confirmed as DENV-1 dengue by the laboratory of Ningbo Municipal Center for Disease Control and Prevention (CDC). The first case was a 6-year old female who developed fever on 11 August. The second one was a 38-year old male whose onset date was on 15 August and his initial symptom was also fever. The two index cases were both connected to community A and had no travel history within 14 days before symptoms onset. These findings revealed that dengue virus had been transmitted among people in the community. By performing a standardized case-finding and field epidemiological investigation, the outbreak source was confirmed as a 65-year old male case living in community B where is about 500 meters far from community A whose onset date was on 30 July. He had the same E gene sequence as the two cases. Therefore, the outbreak provides a typical field to research the disease transmission in a non-epidemic area.

Mathematical model is commonly adopted to explore the transmission of an infectious disease. The basic dengue model is the simple vector-host transmission model in which the host population is represented by a Susceptible–Infectious–Removed (SIR) model and the vector is assumed to remain infectious until death (SI model) [8, 9]. In other researches, internal and external incubation were also considered and the vector and host transmission models were changed to Susceptible–Exposed–Infectious–Removed (SEIR) model and SEI model [10–12]. This study aimed to investigate a dengue outbreak in a non-epidemic area in Ningbo City in China by combining mathematical models we developed, to inspect the epidemiological and virological characteristics of the transmission, and to assess the effectiveness of intervention implemented in the outbreak.

## Methods

### Ethics statement

This effort of outbreak control and investigation was part of Ningbo Municipal CDC’s routine responsibility; therefore, institutional review and informed consent were not required for this study. All data analyzed were anonymized.

### Study design

We performed a time series study in dengue cases reported in Ningbo City from January 2005 to November 2018. We performed a field epidemiological and modelling study to investigate the epidemiological and virological characteristics of a dengue outbreak, to simulate the incidence of the transmission, and to assess the effectiveness of intervention implemented in the outbreak from August to September 2018 in the city. To explore the seasonality patterns of the vector, a surveillance to investigate the density of the vector was also conducted from January to December 2018.

### Study site

Ningbo City (28°51ʹ to 30°33ʹN, 120°55ʹ to 122°16ʹE), locating in the middle section of east coastline and having a population of more than 8 million, is a large city in Zhejiang Province, China. The city has an area of 3730 square kilometers and includes six districts, two counties, and two county-level cities. The ten subareas include 704 communities and 2519 villages. The climate is subtropical monsoon with a yearly average temperature of 16.2°C and a yearly rainfall of 1300 mm to 1400 mm. The rainfall occurs mostly from May to September. According to the dengue surveillance data from 2005 to 2018, Ningbo City is a non-epidemic area in China during the past decade. *Aedes albopictus* was the only vector species in the city. The study community (community A), which has a population of 5536 and an area of about 150 thousand square meters, is a relative separate community in Ningbo City.

### Case definition and case-finding

Case-finding and epidemiological investigation was conducted among all households in the community, the possible clinics, and hospitals based on the following case definition:

- suspected case: a person lived in the community and had got fever symptom since July 25, 2018 (since the onset date of the two index cases was on August 11 and 15, respectively), along with one or more of the following symptoms: rash, headache, muscle pain or joint pains, eyepit pain, facial flush or red skin on the chest, conjunctival injection, or mucosal bleeding; no specific diagnosis was confirmed as other disease.
- clinically diagnosed case: a person who was diagnosed as suspected case and was tested having reduction of white blood-cell counts and platelet counts (lower that 100 × 10^9^/L).
- confirmed case: a person who was diagnosed as suspected case and had one of the following tested results: ①the gene sequence was tested positive by reverse transcription PCR (RT-PCR) or real-time fluorescent quantitative PCR; ②serum specific antibody titers increased more than 4 times in convalescence than in acute phase.

### Vector investigation

Human biting rate (HBR) and larvae density were conducted during the outbreak. In our study, HBR was monitored every day in three surveillance sites locating in the community using the human-baited double net (HDN) trap from August 18, 2018 to the end of the outbreak. One local volunteer was employed and rested inside the small bed net (length × width × height: 1.2m × 1.2m × 2.0m) and was consequently fully protected from mosquitoes during the survey. A larger bed net (length × width × height: 1.8m × 1.8m × 1.5m) was hung over the smaller net and was raised 50 cm above the ground. Both nets were protected from the elements by plastic-sheeting roofs but were not treated with any insecticide. One trained person helped to capture the mosquitoes for 30 minutes in and out the larger bed net per hour during high activity time. The vector species were identified in laboratory and the number of captured mosquitoes was recorded in each survey site to calculate the HBR (per person per hour).

We used Breteau Index (BI) to assess the larvae density of the vector. An investigation of BI was conducted from 18 August to 14 September in the community. More than 100 households were surveyed each time by screening all possible habitats and larvae or pupae of the vector were collected and numbered to bring back to the laboratory of Ningbo Municipal CDC to bleed them until they emerged to adult mosquitoes for species classification. Therefore, BI was calculated per 100 households in each day of the community.

### Vector seasonality surveillance data in 2018

To identify the density and the seasonality of the vector in the city, the density of adult and larvae vector was monitored each month in 2018 from January to December. The adult density was monitored by using light-trap density. More than four surveillance sites were selected in each subarea of Ningbo City, among which two were selected in urban area such as parks and two were in villages’ households. A light-trap was hung about 1.5m above the ground in each mosquito capturing site from one hour before sunset to one hour after sunrise of the next day. *Aedes* mosquitoes were sorted and identified according to morphological characteristics. The light-trap density of the vector was calculated per light-trap per night consequently. BI was also monitored each month in four communities or villages. The investigation method was the same as the one used during the outbreak.

### Case specimen collection and gene sequence analysis of the virus

A blood specimen was collected from each case and was sent to the laboratory of Ningbo Municipal CDC by cold chain and stored at -80°C freezer immediately in preparation for identification using PCR and gene sequencing.

Nucleic acids were extracted using TGuide S32 Magnetic Viral DNA&RNA Kit (TIANGEN BIOTECH [BEIJING] Co., Ltd, Beijing, China), according to the manufacturer’s instructions. RT-PCR was used to detect the universal and specific primer. The primer sequence and the test procedure were performed according to “Diagnosis for dengue fever (WS 216–2018)” announced by National Health Commission of the People’s Republic of China. The E gene of dengue virus was amplified using one Step RNA PCR Kit (TaKaRa [Dalian] Co., Ltd, Dalian, China), according to the manufacturer’s instructions. The PCR procedures were performed as the protocol of E gene amplification which was deposited in protocols.io (dx.doi.org/10.17504/protocols.io.zpuf5nw). The purified products amplified by PCR were used for gene sequencing directly. The sequencing was performed by Sunny Co., Ltd, Shanghai, China. The phylogenetic trees for the E genes were constructed using the neighbor-joining method provided by MEGA 5.2 software. Nucleotides and amino acids similarity were compared by MegAlign software.

### Countermeasures implemented in the outbreak

Case isolation and treatment for all cases was implemented immediately on 18 August, 2018. From 19 August, outbreak control strategy was implemented including household survey, case-finding, health promotion, environmental cleaning, adult vector control by applying insecticides as residual spraying and space spraying in the morning and at dusk, and surveillance of BI and HBR every day during the outbreak. For case isolation, all cases (including suspected cases, clinically diagnosed cases, and confirmed cases) were quarantined individually in hospitals until 5 days after illness onset date or until all the symptoms disappeared after 24 hours for those whose symptoms lasted longer than 5 days. The public health professionals performed active case finding in household survey every day according to the case definition.

### Dynamics model with no intervention

**Fig 1:**
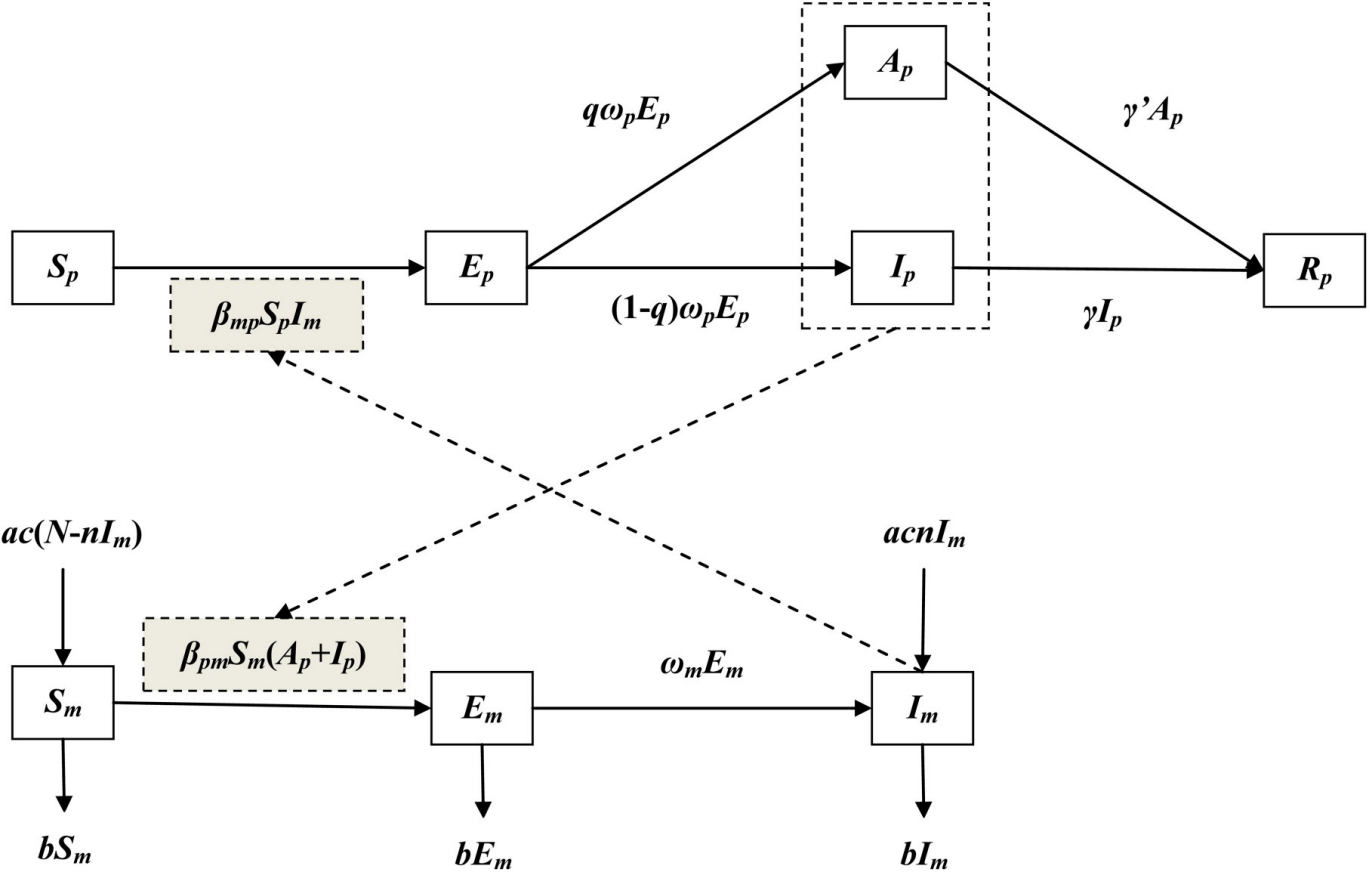
Flowchart of development of the dengue transmission model.

Several ordinary differential equation (ODE) models were developed to simulate the transmission of the virus [11–13]. In this study, the transmission model was developed based our previous study[14]. In the model, individuals were divided into the following six compartments: *S*_*p*_, susceptible; *E*_*p*_, exposed; *I*_*p*_, infectious; *A*_*p*_, asymptomatic; *R*_*p*_, removed. Vectors were divided into the following three compartments: *S*_*m*_, susceptible; *E*_*m*_, exposed; *I*_*m*_, infectious. Therefore, total number of the vectors (*N*_*m*_) was calculated by adding up the three mosquito compartments. The human and vector compartments interacted with each other according to Fig 1.

The model (Model 1) was therefore shown as follows:

$$\frac{dS_{p}}{dt}=-\beta_{mp}S_{p}I_{m}$$

$$\frac{dE_{p}}{dt}=\beta_{mp}S_{p}I_{m}-\omega_{p}E_{p}$$

$$\frac{dI_{p}}{dt}=(1-q)\omega_{p}E_{p}-\gamma I_{p}$$

$$\frac{dA_{p}}{dt}={q\omega }_{p}E_{p}-\gamma^{\prime }\;A_{p}$$

$$\frac{dR_{p}}{dt}={\gamma I}_{p}+{\gamma^{\prime }\;A}_{p}$$

$$\frac{dS_{m}}{dt}=ac(N_{m}-nI_{m})-\beta_{pm}S_{m}(A_{p}+I_{p})-bS_{m}$$

$$\frac{dE_{m}}{dt}=\beta_{pm}S_{m}(A_{p}+I_{p})-(b+\omega_{m})E_{m}$$

$$\frac{dI_{m}}{dt}=acnI_{m}+{\omega_{m}E}_{m}-bI_{m}$$

$$N_{m}=S_{m}+E_{m}+I_{m}$$

In the equations, 11 parameters (*β*_*mp*_, *β*_*pm*_, *ω*_*p*_, *ω*_*m*_, *q*, *γ*, *γ*’, *a*, *c*, *n*, and *b*) are included. The definitions of the parameters are summarized in Table 1.

**Table 1:** Parameter definitions and values.

Parameter	Description	Unit	Value	Range	Method
*β*_*mp*_	Transmission relative rate from mosquitos to human	1	1.4000	≥ 0	Curve fitting
*β*_*pm*_	Transmission relative rate from human to mosquitos	1	1.3613	≥ 0	Curve fitting
*ω*_*p*_	Incubation relative rate of human infection	day^-1^	0.1667	0.1250–0.2500	References [11]
*ω*_*m*_	Incubation relative rate of mosquitos infection	day^-1^	0.1000	0.0833–0.1250	References [13]
*q*	Proportion of human asymptomatic infection	1	0.6875	0–1	References [15]
*γ*	Removed relative rate of infectious individuals	day^-1^	0.1429	0.0714–0.3333	References [12, 13]
*γ*’	Removed relative rate of asymptomatic individuals	day^-1^	0.1429	0.0714–0.3333	References [12, 13]
*a*	Daily birth rate of mosquitos	day^-1^	0.0714	0.0200–0.2500	References [13]
*c*	Seasonality parameter of the mosquitos population	1	See text*	0–1	Curve fitting
*τ*	Simulation delay of the initial time in the whole season	day	170	≥ 0	Analysis on the reported data
*T*	Duration of the cycle	day	712	≥ 0	Analysis on the reported data
*x*	Changing rate coefficient of the birth rate	1	-0.6200	≤ 0	Curve fitting
*n*	Proportion of transovarial transmission	1	0.1000	0.0140–0.1740	References [16]
*b*	Daily death rate of mosquitos	day^-1^	0.0714	0.0200–0.2500	References [13]
*j*	Changing rate coefficient of the density	1	-1.1310	≤ 0	Curve fitting
*θ*	Effective coefficient of rHBR	1	0–0.9	0–1	Simulated

* the parameter *c* was simulated by the trigonometric function using parameters *τ* and *T*.

According to the published researches, seasonality could be simulated by trigonometric function. Therefore, we assumed that the function of parameter *c* was as follows:

$$c=\cos\left[\frac{2\pi (t-\tau )}{T}\right]$$

In the model, *τ* and *T* refer to simulation delay of the initial time in the whole season and the duration of the season, respectively. Model 1 was therefore changed to Model 2 which includes the seasonality of vectors and was shown as follows:

$$\frac{dS_{p}}{dt}=-\beta_{mp}S_{p}I_{m}$$

$$\frac{dE_{p}}{dt}=\beta_{mp}S_{p}I_{m}-\omega_{p}E_{p}$$

$$\frac{dI_{p}}{dt}=(1-q)\omega_{p}E_{p}-\gamma I_{p}$$

$$\frac{dA_{p}}{dt}={q\omega }_{p}E_{p}-\gamma^{\prime }\;A_{p}$$

$$\frac{dR_{p}}{dt}={\gamma I}_{p}+{\gamma^{\prime }\;A}_{p}$$

$$\frac{dS_{m}}{dt}=a\;\cos\left[\frac{2\pi (t-\tau )}{T}\right](N_{m}-nI_{m})-\beta_{pm}S_{m}(A_{p}+I_{p})-bS_{m}$$

$$\frac{dE_{m}}{dt}=\beta_{pm}S_{m}(A_{p}+I_{p})-(b+\omega_{m})E_{m}$$

$$\frac{dI_{m}}{dt}=a\;\cos\left[\frac{2\pi (t-\tau )}{T}\right]nI_{m}+{\omega_{m}E}_{m}-bI_{m}$$

$$N_{m}=S_{m}+E_{m}+I_{m}$$

### The model of case isolation

Case isolation was implemented when the case was confirmed. With the intervention, the contact between the case and mosquitos was halted. However, asymptomatic infection is commonly hard to find out because there is no symptom of the infection for the surveillance. Therefore, in this condition, the transmission still exists from asymptomatic individuals to the vectors and from the vectors to human. The model (Model 3) of case isolation is as follows:

$$\frac{dS_{p}}{dt}=-\beta_{mp}S_{p}I_{m}$$

$$\frac{dE_{p}}{dt}=\beta_{mp}S_{p}I_{m}-\omega_{p}E_{p}$$

$$\frac{dI_{p}}{dt}=(1-q)\omega_{p}E_{p}-\gamma I_{p}$$

$$\frac{dA_{p}}{dt}={q\omega }_{p}E_{p}-\gamma^{\prime }\;A_{p}$$

$$\frac{dR_{p}}{dt}={\gamma I}_{p}+{\gamma^{\prime }\;A}_{p}$$

$$\frac{dS_{m}}{dt}=a\;\cos\left[\frac{2\pi (t-\tau )}{T}\right](N_{m}-nI_{m})-\beta_{pm}S_{m}A_{p}-bS_{m}$$

$$\frac{dE_{m}}{dt}=\beta_{pm}S_{m}A_{p}-(b+\omega_{m})E_{m}$$

$$\frac{dI_{m}}{dt}=a\;\cos\left[\frac{2\pi (t-\tau )}{T}\right]nI_{m}+{\omega_{m}E}_{m}-bI_{m}$$

$$N_{m}=S_{m}+E_{m}+I_{m}$$

### The model of reducing HBR

To reducing the HBR (rHBR) during the outbreak, bed net or mosquito repellents were encouraged to be used in the community. We assumed that the transmission relative rate from mosquitos to human and from human to mosquitos would be multiplied by an effective coefficient *θ* when the HBR was reduced by different levels (10% to 100%). Therefore, *θ* would be set as 0.9, 0.8, 0.7, …, and 0.0 consequently.

### The model of vector control

In this study, adult vector control (AVC) and larvae control (LC) were simulated. For AVC, adult vector were controlled by applying insecticides as space spraying during the outbreak. In this condition, the density of adult vector would decline sharply following an exponential model:

$$N_{m}=j_{0}e^{jt}$$

In the exponential model, *j*_0_ and *j* refer to the baseline (*t* = 0) and the changing rate coefficient of the density. The reduction of the density is actually resulted from the changing of lifetime parameter *b* (*b*_*t*_ *= b*_0_ *+ j*). The model (Model 4) of AVC is as follows:

$$\frac{dS_{p}}{dt}=-\beta_{mp}S_{p}I_{m}$$

$$\frac{dE_{p}}{dt}=\beta_{mp}S_{p}I_{m}-\omega_{p}E_{p}$$

$$\frac{dI_{p}}{dt}=(1-q)\omega_{p}E_{p}-\gamma I_{p}$$

$$\frac{dA_{p}}{dt}={q\omega }_{p}E_{p}-\gamma^{\prime }\;A_{p}$$

$$\frac{dR_{p}}{dt}={\gamma I}_{p}+{\gamma^{\prime }\;A}_{p}$$

$$\frac{dS_{m}}{dt}=a\;\cos\left[\frac{2\pi (t-\tau )}{T}\right](N_{m}-nI_{m})-\beta_{pm}S_{m}(A_{p}+I_{p})-(b_{0}+j)S_{m}$$

$$\frac{dE_{m}}{dt}=\beta_{pm}S_{m}(A_{p}+I_{p})-(b_{0}+j+\omega_{m})E_{m}$$

$$\frac{dI_{m}}{dt}=a\;\cos\left[\frac{2\pi (t-\tau )}{T}\right]nI_{m}+{\omega_{m}E}_{m}-(b_{0}+j)I_{m}$$

For LC, environmental management and modification, disposing of solid waste properly and removing artificial man-made habitats, covering, emptying and cleaning of domestic water storage containers, were adopted to prevent mosquitoes from accessing egg-laying habitats. Under this circumstance, the birth rate from egg to adult vector would decline sharply following an exponential model:

$$a_{t}=a_{0}e^{xt}$$

In the exponential model, *a*_0_ and *x* refer to the baseline (*t* = 0) and the changing rate coefficient of the birth rate. The model (Model 5) of LC is as follows:

$$\frac{dS_{p}}{dt}=-\beta_{mp}S_{p}I_{m}$$

$$\frac{dE_{p}}{dt}=\beta_{mp}S_{p}I_{m}-\omega_{p}E_{p}$$

$$\frac{dI_{p}}{dt}=(1-q)\omega_{p}E_{p}-\gamma I_{p}$$

$$\frac{dA_{p}}{dt}={q\omega }_{p}E_{p}-\gamma^{\prime }\;A_{p}$$

$$\frac{dR_{p}}{dt}={\gamma I}_{p}+{\gamma^{\prime }\;A}_{p}$$

$$\frac{dS_{m}}{dt}=a_{0}e^{xt}\;\cos\left[\frac{2\pi (t-\tau )}{T}\right](N_{m}-nI_{m})-\beta_{pm}S_{m}(A_{p}+I_{p})-bS_{m}$$

$$\frac{dE_{m}}{dt}=\beta_{pm}S_{m}(A_{p}+I_{p})-(b+\omega_{m})E_{m}$$

$$\frac{dI_{m}}{dt}=a_{0}e^{xt}\;\cos\left[\frac{2\pi (t-\tau )}{T}\right]nI_{m}+{\omega_{m}E}_{m}-bI_{m}$$

In addition, vector control activity was launched to improve community participation and mobilization for sustained vector control; to carry out the monitoring and surveillance of the vector as a routine work.

### Parameter estimation

There were 15 parameters, *β*_*mp*_, *β*_*pm*_, *ω*_*p*_, *ω*_*m*_, *q*, *γ*, *γ*’, *a*, *c*, *n*, *b*, *τ*, *T*, *j* and *x*, in the models (Table 1). Symptoms of infection usually begin 4–8 days after the mosquito bite [11]. Here we simulated 6 days of the incubation period in our model, thus *ω*_*p*_ = 0.1667. After entering the mosquito in the blood meal, the virus will require an additional 8–12 days incubation before it can then be transmitted to another human [13]. Here we simulated 10 days in our model, thus *ω*_*m*_ = 0.1000. The ratio of symptomatic compared to asymptomatic infection is 2.2:1 [15], thus *q* = 0.6875. The infectious period is ranging from 3 days to 14 days [12, 13]. Here we simulated 7 days in our model, thus *γ* = *γ*’ = 0.1429. *Aedes albopictus* appears to be an efficient vertical transmitter of DENV-1 [16]. Vertical infection rates (the percentage of offspring vertically infected) of individual positive families are ranging from 1.4% to 17.4% [16]. Here we simulated 10.0% in our model, thus *n* = 0.1000. The mosquito remains infected for the remainder of its life, which might be from 4 days to 50 days [13]. Since the outbreak occurred in August, we simulated 14 days in our model, thus *b* = 0.0714. Since we simulated the transmission in August when the density of the vector was at the peak, which means that the vector has a balance population and the birth rate is equal to the death rate, thus *a* = *b* = 0.0714. However, the vector population decreased after August and the two parameters were adjusted by the seasonality equation accordingly.

Parameters *β*_*mp*_ and *β*_*pm*_ were calculated using the Models 1–5 to fit the reported cases data. The seasonality relative parameter *τ* and *T* were confirmed according to the onset date of the first case, the duration of the season, and curve fitting to the light-trap captured density and BI surveillance data in 2018. Thus *c* was simulated consequently.

Adult mosquito control parameter *j* was estimated by the exponential model fitted with the HBR surveillance data during the outbreak. Larvae control parameter *x* was estimated by the exponential model fitted with the BI surveillance data during the outbreak.

To quantify the significance of the curve fitting, the chi-square test was employed by using a significance level of 0.05, which means that the model fits the reported data well when *P* > 0.05. The value of chi-square was calculated by the following equations:

$$\chi^{2}=\frac{{\left(A_{r}-T_{s}\right)}^{2}}{T_{s}}$$

In the equation, *A*_*r*_ and *T*_*s*_ refer to reported data and simulated data, respectively.

### Indicators for assessing the effectiveness of interventions

Absolute effectiveness (AE) and relative effectiveness of the interventions were calculated by the following equations:

$$AE={TAR}_{1}-{TAR}_{2}$$

$$RE=\frac{{TAR}_{1}-{TAR}_{2}}{{TAR}_{1}}\times 100\%$$

In the equations, *TAR*_1_ and *TAR*_2_ refer to the total attack rate (TAR) without and with intervention, respectively.

### Simulation method and statistical analysis

A two-step simulation method was adopted. The first simulation step was to model the transmission source from community B to community A. The transmission source from community B is an imported case to community A. The importation can be simulated by using an impulse function according to our published research [17]. In this step, transmission source is modeled by the following impulse function:

$$transmission\;source=impulse(n,t_{0},t_{i})$$

In the function, *n*, *t*_0_ and *t*_*i*_ refer to number of dengue cases, start time of the simulation, and the interval of the transmission source. Considering that there was only one dengue case in community B during the outbreak, we simulated that a dengue case impulse into community A on 30 July, 2018. Therefore, *n* = 1, *t*_0_ = 0, *t*_*i*_ = ∞.

The second step simulation was to model the transmission in community A using Models 1–5. Berkeley Madonna 8.3.18 (developed by Robert Macey and George Oster of the University of California at Berkeley) was employed for model simulation and least root mean square was adopted to judge the goodness of fit of curve fitting between the simulation models and the reported data. The simulation methods were the same as the previously published researches [17–21].

The chi-square test, which was adopted to quantify the significance of the goodness of fit, was performed by SPSS 13.0 (IBM Corp., Armonk, NY, USA).

### Sensitivity analysis

Since 8 parameters (*ω*_*p*_, *ω*_*m*_, *q*, *γ*, *γ*’, *a*, *n*, and *b*) were estimated by references, there might be some uncertainty about them which might impact the results of models we built. In our study, sensitivity analysis was performed by varying the 8 parameters based on the methods we adopted in our previous research [18]. The ranges of the 8 parameters, which were shown in Table 1, were split into 1000 values and simulated separately. The prevalence of each simulation were used to calculate the mean prevalence, mean–sd, and mean + sd.

## Results

### Reported cases in the city from 2005 to 2018

**Fig 2:**
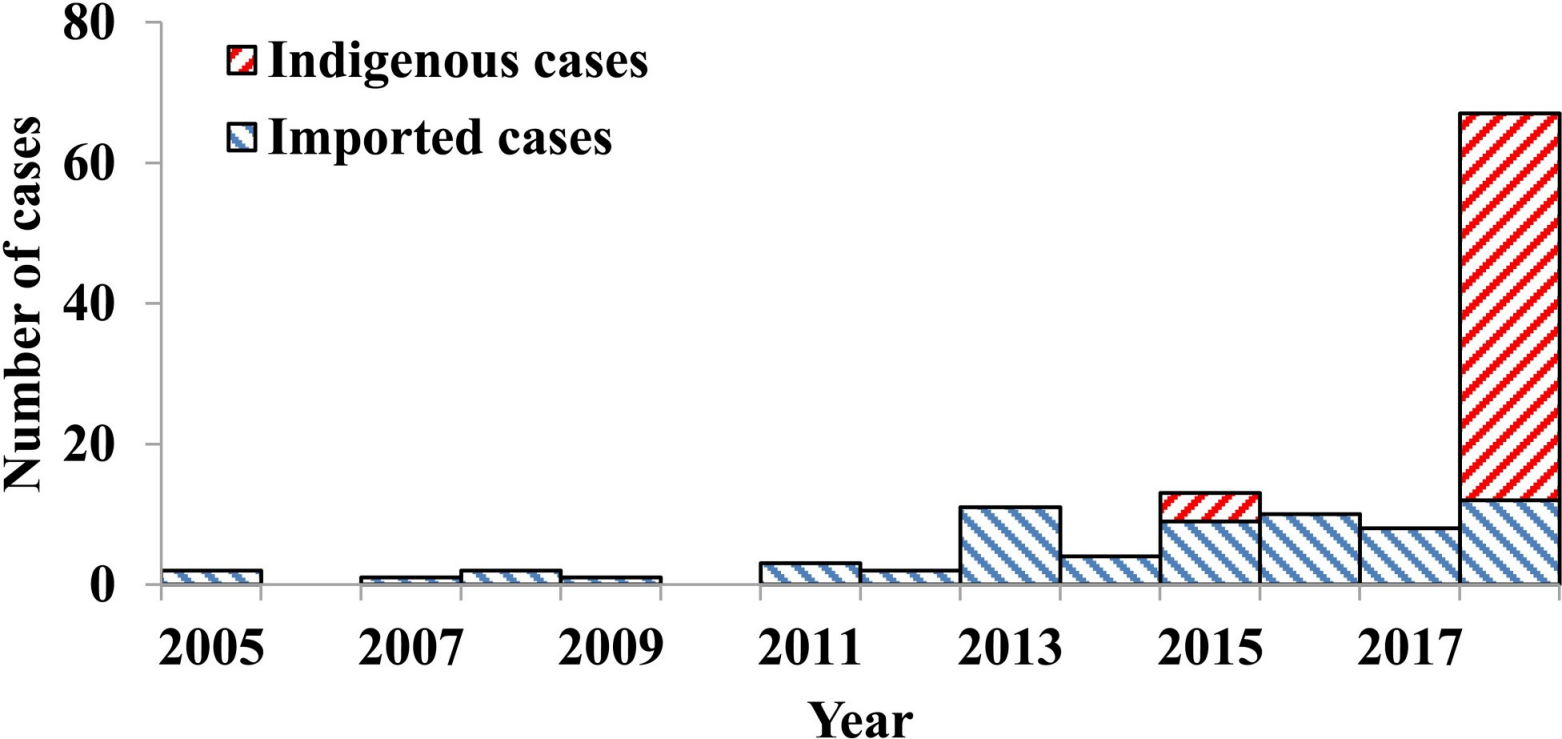
Reported dengue cases from 2005 to 2018 in Ningbo City, China.

There were totally 124 dengue cases reported in Ningbo City from January 2005 to November 2018, of which 52.42% (65/124) were imported cases. No indigenous case was reported during 2005 to 2014. However, 4 indigenous cases were confirmed in 2015. After that year, 55 more indigenous cases were confirmed till the end of 2018 (Fig 2).

### Epidemiological characteristics of the outbreak

**Fig 3:**
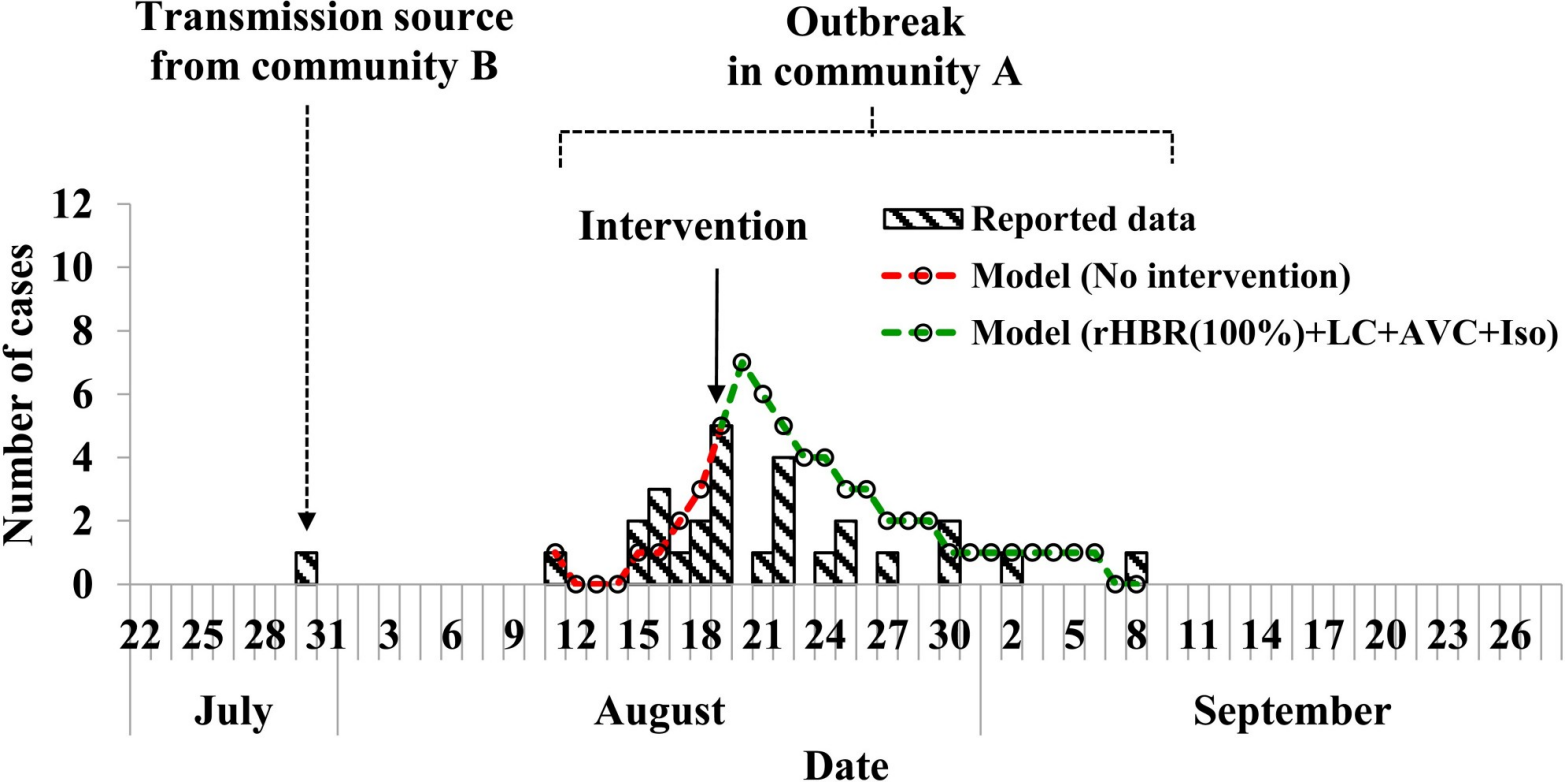
Epidemic curve and curve fitting of a dengue outbreak in a community in Ningbo City, China in 2018.

There was a dengue outbreak happened in 2018, with totally 27 indigenous cases confirmed by PCR method. The index case was reported on 11 August, 2018. Cases increased gradually and reached a peak on 19 August. As an integrated control intervention was implemented focusing on case isolation, adult and larva mosquito control, the incidence decreased accordingly. The last reported case was on 8 September, 2018. The total attack rate (TAR) of the outbreak was 0.49% (Fig 3).

Among these cases, 12 were male while 15 female cases with age range 6–86 years. The age and sex distribution of the cases was shown in Table 2.

**Table 2:** Age and sex distribution of the cases during the outbreak in 2018.

Variables	Number of cases	Percentage (%)
Age (Year)		
< 10	1	3.70
10 -	1	3.70
20 -	2	7.41
30 -	2	7.41
40 -	5	18.52
50 -	6	22.22
60 -	5	18.52
> 70	5	18.52
Gender		
Male	12	44.44
Female	15	55.56

By performing a standardized case-finding and field epidemiological investigation, the outbreak source was confirmed as a 65-year old male case living in community B where is about 500 meters far from community A. The case developed symptoms on 30 July, 2018 and only visited the hospital on 3 August and was confirmed as a dengue fever case by PCR method. The case had visited community A several times after disease onset and before hospital diagnosis.

### Laboratory analysis

**Fig 4:**
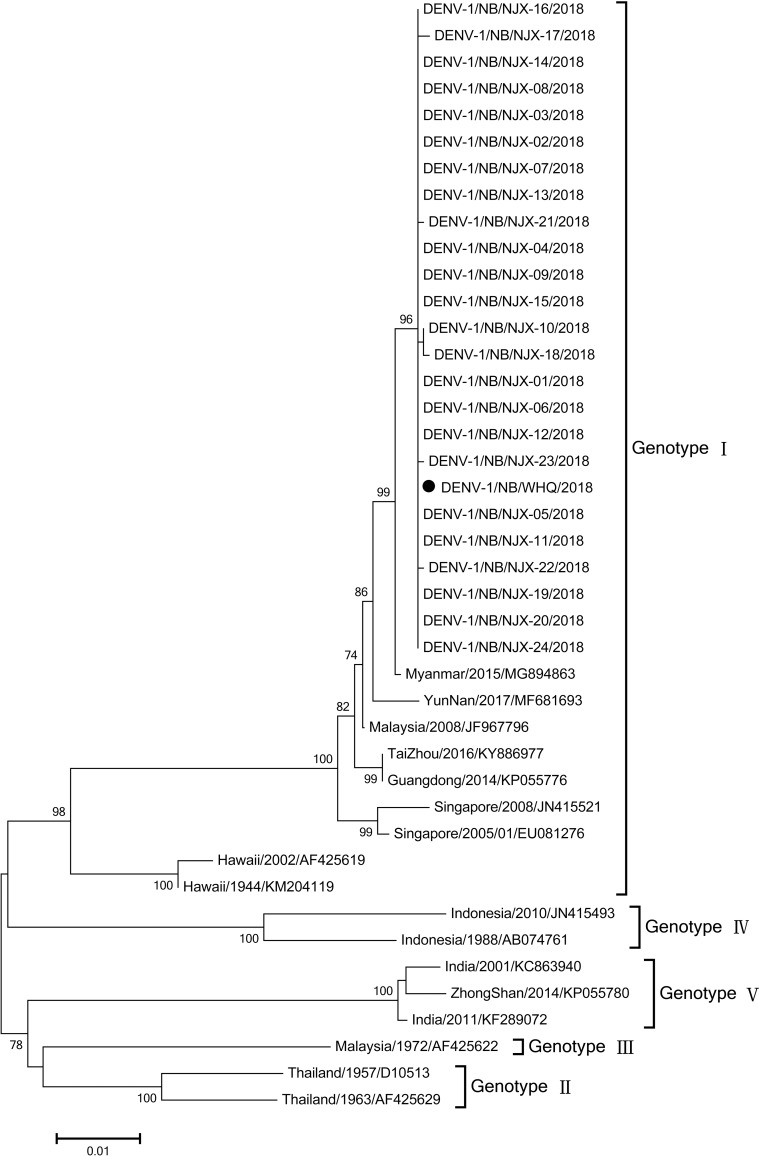
Phylogenetic analysis of E genes of the 25 GENV-1 viruses isolated from 25 patients in a dengue outbreak in Ningbo City, China in 2018.

Of the 28 cases in communities A and B, specimens of 25 cases were obtained to detect the whole E gene sequences (S1 Table). The length of the sequence was 1485bp which coded 495 amino acids. Compared with the gene sequences in the GenBank database, the gene type of all 25 cases were all DENV-1. Phylogenetic analysis revealed that 25 virus strains in the outbreak highly aggregated into a branch which belonged to Genotypes I (GI) and were closely related to the virus in community B and the seven strains in Asia countries including China (YunNan, TaiZhou, and Guangdong), Myanmar, Malaysia, and Singapore, were closely related to the prototype strain Myanmar/2015/MG894863 which was origin from Myanmar and export to Taiwan Province, China in 2015, however they were much far from the prototype strain Hawaii/1944/KM204119 (Fig 4). The names and the GenBank accession numbers of the 25 viruses were shown in S1 Table. The all 25 isolates shared high levels of nucleotide identity and amino acid similarity with the seven GI reference strains: 98.0%– 99.7% and 96.6%– 98.2%, respectively, while moderate levels with the prototype strain Hawaii/1944/KM204119: 94.5% –94.8% and 92.1% –92.5%, respectively and low levels with the prototype strains of Genotypes II–IV (G II–IV).

With both the epidemiological evidence and the gene sequencing results, we concluded that the case that was found in community B was the transmission source of the outbreak in community A.

### Model fitting

**Fig 5:**
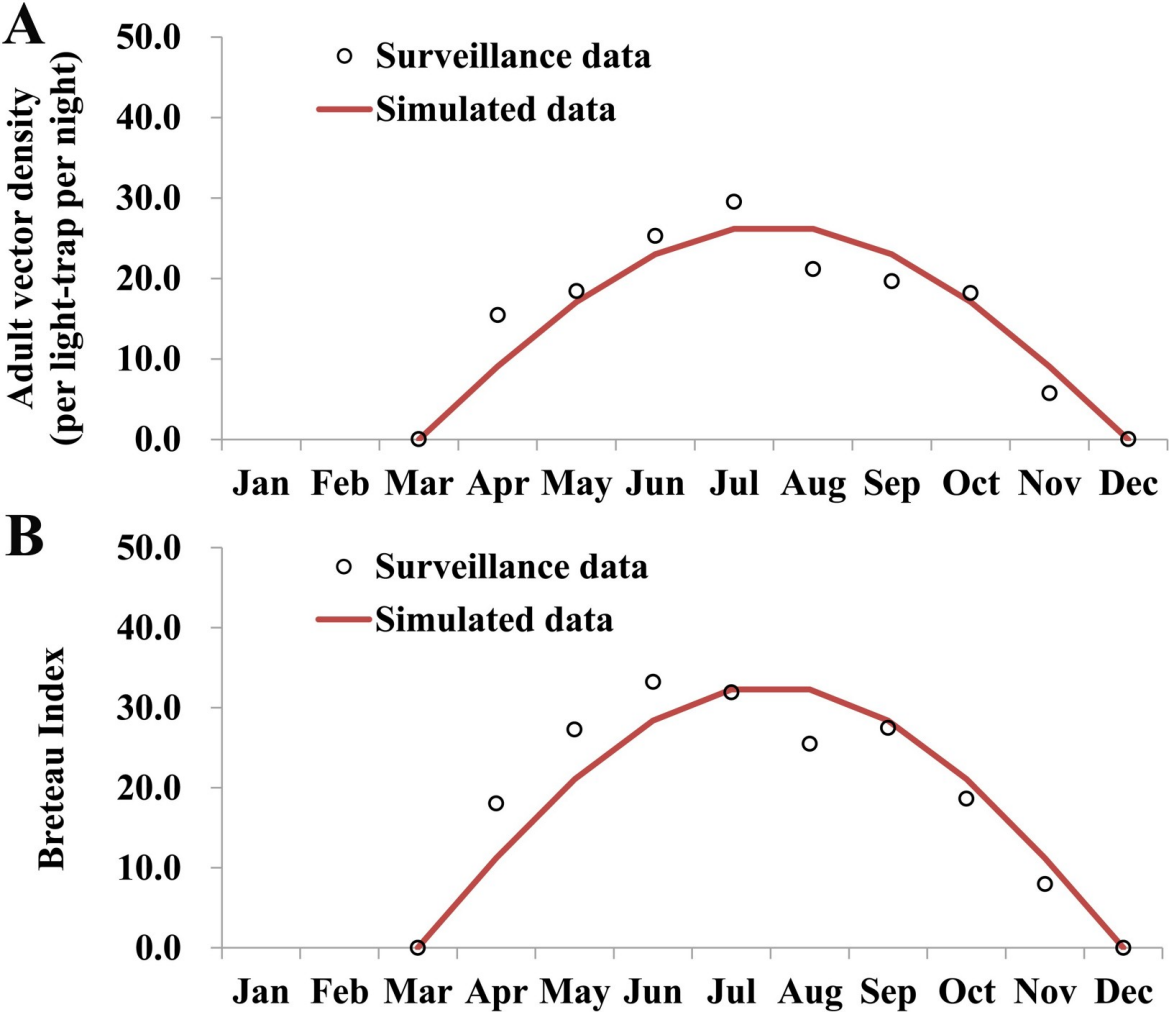
Curve fitting of adult and larvae surveillance data in Ningbo City, 2017. A, monthly adult density of the vector; B, monthly Breteau Index.

According to the surveillance data of adult vector in 2018, the median monthly density of the vector was 18.30 per light-trap per night with a range of 0.00 to 29.49. The peak light-trap density of the vector occurred in July. No statistically significance was observed by the curve fitting (*χ*^2^ = 7.996, *P* = 0.434), thus the seasonality of the density (Fig 5A) could be simulated by the following sine function:

$$Density=26.56\;\sin\left[\frac{2\pi (t-2)}{18}\right]$$

The median monthly BI was 22.08, with a range of 0.00 to 33.22. The peak light-trap density of the vector occurred in June. The seasonality of BI (Fig 5B) could be simulated well (*χ*^2^ = 9.46, *P* = 0.305) by the following sine function:

$$BI=32.79\;\sin\left[\frac{2\pi (t-2)}{18}\right]$$

Consequently, the seasonality of the vector population dynamic could be simulated by a trigonometric function with a cycle of 18 months. From Fig 5, we can see that the model simulated 10 months (306 days) in a year, thus, *T* = 712. As the illness onset date was on 11 August, 2018, the simulation delay of the initial time in the whole season is 170 days, thus *τ* = 170.

By fitting with the reported cases data (S1 Fig), Model 1 ran well (*χ*^2^ = 6.324, *P* = 0.388) and reported the optimal values of *β*_*mp*_ and *β*_*pm*_ which were 1.4000 and 1.3613, respectively.

The results of sensitivity analysis showed that the model is not sensitive to the parameters *ω*_*m*_, *a*, and *n*. Our model is slightly sensitive to the parameter *ω*_*p*_, but sensitive to *q*, *γ*, *γ*’, and *b* (S2 Fig).

### Effectiveness of the interventions

On 18 August, 2018, BI and HBR were 57.5 and 12 per person per hour. After that, BI and HBR decreased dramatically because of the implementation of the vector control interventions. BI could be simulated by the following exponential model:

$$BI=57.5e^{-0.6200t}$$

HBR could be simulated by the following exponential model:

$$HBR=12e^{-1.1310t}$$

**Fig 6:**
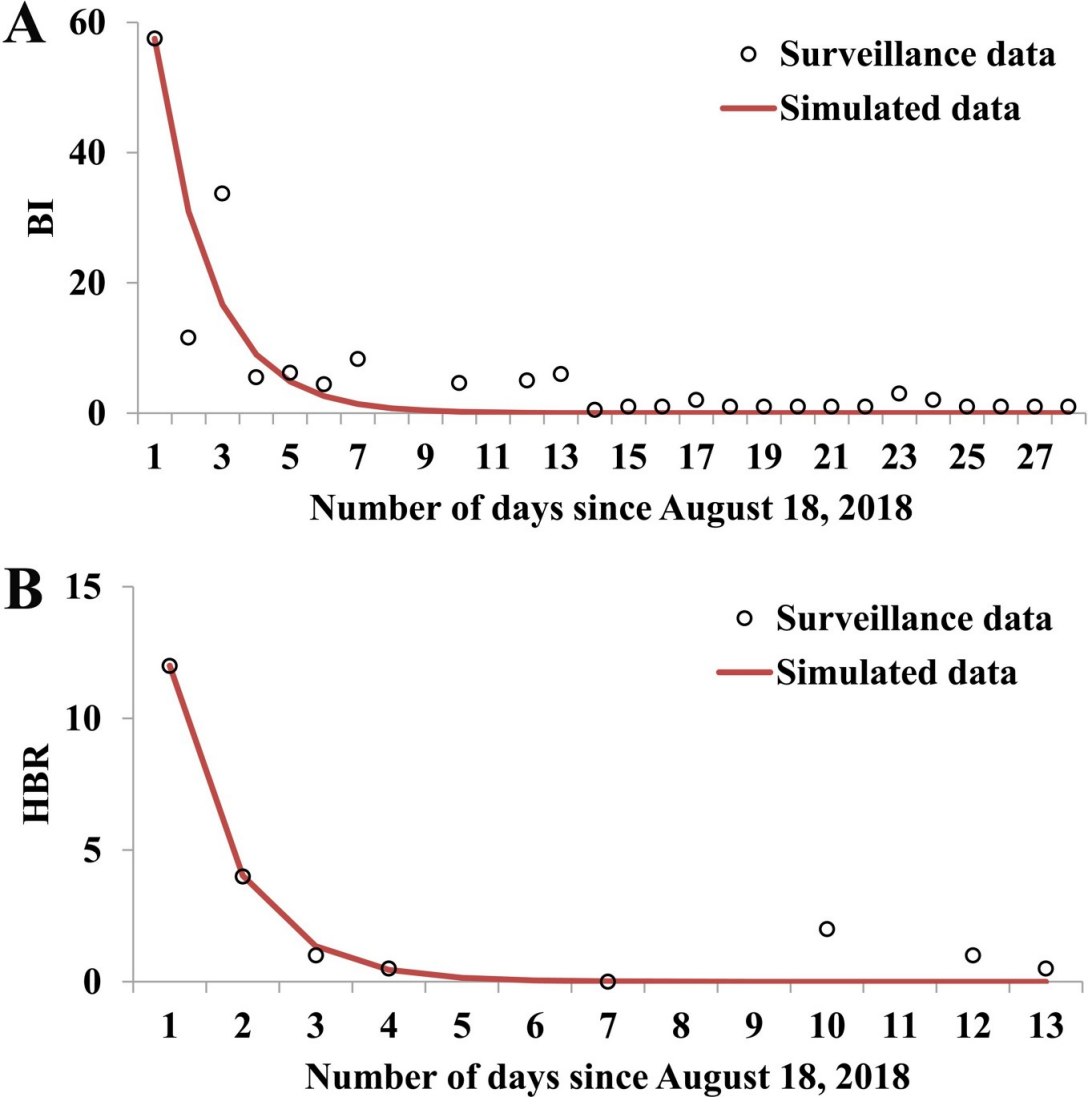
Curve fitting of BI and HBR data during the dengue outbreak in Ningbo City, 2018. A, daily Breteau Index (BI); B, daily human biting rate (HBR).

The Malthusian models and surveillance data were shown in Fig 6. According to the simulation model with no intervention, the total number of the cases would be 1728 with a TAR of 31.21% (95%CI: 29.99%– 32.43%). This result revealed that the AE and RE of the integrated outbreak control strategy implemented by Ningbo Municipal CDC were 30.72% and 98.44%, respectively.

As shown in Table 3, case isolation and LC implemented from 11 August has almost the same TAR and DO as no intervention.

**Table 3:** Results of simulated effectiveness of dengue interventions implemented on August 11, 2018.

Intervention	Number of cases	TAR (%)		AE (%)	RE (%)	DO (Days)
		%	95% CI			
No intervention	1728	31.21	29.99–32.43	0.00	0.00	57
Iso	1728	31.21	29.99–32.43	0.00	0.00	57
rHBR						
10%	1727	31.20	29.98–32.42	0.01	0.05	57
20%	1727	31.20	29.98–32.42	0.01	0.05	58
30%	1727	31.20	29.98–32.42	0.01	0.05	59
40%	1727	31.20	29.98–32.42	0.01	0.05	60
50%	1728	31.21	29.99–32.43	0.00	0.00	63
60%	1726	31.18	29.96–32.40	0.03	0.10	67
70%	1708	30.85	29.64–32.07	0.36	1.15	80
80%	1475	26.64	25.48–27.81	4.57	14.63	102
90%	567	10.24	9.44–11.04	20.97	67.18	99
100%	58	1.05	0.78–1.32	30.16	96.64	27
LC	1728	31.21	29.99–32.43	0.00	0.00	57
AVC	105	1.90	1.54–2.26	29.31	93.92	32
LC+AVC	105	1.90	1.54–2.26	29.31	93.92	32
rHBR_100%_+AVC	58	1.05	0.78–1.32	30.16	96.64	27
rHBR_100%_+LC	58	1.05	0.78–1.32	30.16	96.64	27
rHBR_100%_+LC+AVC	58	1.05	0.78–1.32	30.16	96.64	27
rHBR_100%_+LC+AVC+Iso	58	1.05	0.78–1.32	30.16	96.64	27
Reported data	27	0.49	0.30–0.67	30.72	98.44	29

Iso, case isolation; rHBR, reducing human biting rate; LC, larvae control; AVC, adult vector control; CI, confidence interval calculated by binomial distribution.

Different levels of rHBR had different effectiveness with TARs ranging from 1.05% to 31.21% and DOs ranging from 27 days to 102 days. The TARs could be simulated by the following logistic differential equation model:

$$\frac{dTAR}{dt}=-2.9380TAR\left(1-\frac{TAR}{31.22}\right)$$

**Fig 7:**
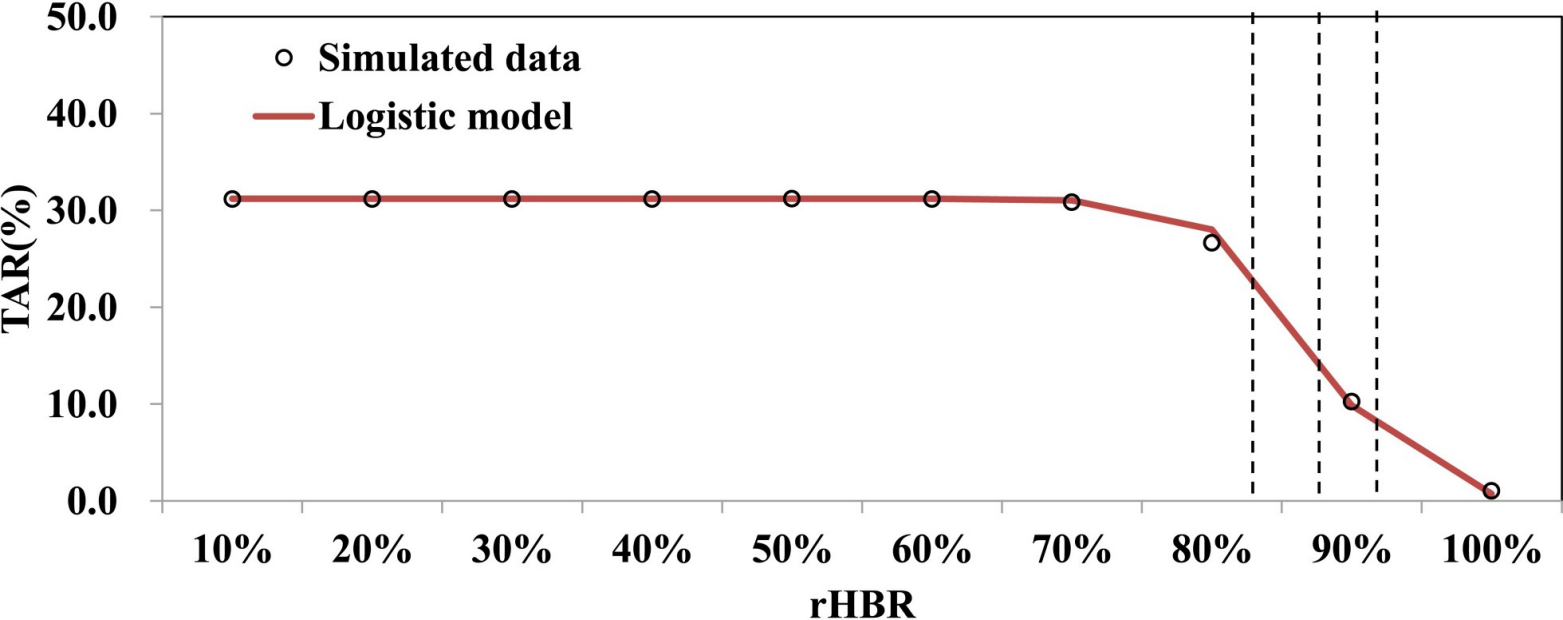
Curve fitting of logistic model and TARs data at different levels of rHBR.

The logistic model fitted the data well (*χ*^2^ = 0.199, *P* = 1.000) and showed that the TAR began decreased quickly when rHBR was higher than 82.8% but decreased slowly when rHBR was higher than 91.8% (Fig 7).

AVC had a very low TAR and DO. Combined-intervention strategy that consists of LC and AVC had a similar TAR and DO as that of AVC. Combined-intervention strategy that consists of “rHBR_100%_+AVC”, “rHBR_100%_+LC”, “rHBR_100%_+LC+AVC”, and “rHBR_100%_+LC+AVC+Iso” had the same effectiveness (TAR, DO, AE, and RE) and had a similar epidemic curve as reported data (Fig 3).

## Discussion

Since 1929, there is only one dengue outbreak happened in 2004 in Ningbo City [22]. We analyze the reported data from 2005 to 2018 and comment that there was no dengue transmission in the city from 2005 to 2014 and the city was a non-epidemic area of dengue virus for more than 10 years. Therefore, by analyzing the epidemiological and the virological characteristics of the first dengue outbreak in a non-epidemic area since 2005 (the second outbreak since 1929), our study can provide important reference for the disease control and prevention.

### The virus and the vector

Although four serotypes of dengue virus were isolated in China [2], DENV-1 has become the predominant serotype since the 1990s [2, 22, 23]. Our gene sequencing findings also showed that the same serotype in the outbreak of community A in Ningbo City. This revealed that DENV-1 may still be the target virus in the future outbreak control.

The virus is transmitted by female mosquitoes mainly by the species *Ae*. *aegypti* and, to a lesser extent, by *Ae*. *albopictus*. However, the published findings of the disease outbreaks showed that the virus was spread mostly through *Ae*. *albopictus* in China, especially in Guangdong Province [2, 6]. The outbreak in Ningbo City revealed that the virus is expanding its transmission area through *Ae*. *albopictus* in China, and that it is an urgent issue to look insight of the transmissibility of the vector and to assess the vector specific countermeasures for disease control and prevention.

The surveillance of BI and light-trap density of the vector showed that the seasonality of vector is obvious with a peak from June to August in the city. The high density of the vector provides a high receptivity and therefore a high probability for the transmission. This is also the case in Ningbo City on 18 August, 2018. These findings of seasonality of vector density and the incidence were similar to transmission pattern of dengue in China, with indigenous cases mainly reported from July to November with the peak transmission mostly in the hot and humid seasons [2].

### Validity of the model

In our study, the ODE models were employed to fit the epidemic curves of the outbreak in a large community, the results of the Chi square test showed high good-of-fitness of our models to the reported data, suggesting that the models is suitable for this study and can be used to simulate the incidence of the outbreak and to assess the effectiveness of the countermeasures. The results of sensitivity analysis showed that our model is sensitive to *q*, *γ*, *γ*’, and *b*. Therefore, more field epidemiological investigation is needed to explore the proportion of asymptomatic infections, the duration from illness onset to recovery, the infectious period, and the mortality rate of the vector.

### The spread of the virus

Our model shows that dengue virus could lead to a high attack rate (higher that 30%) within two months without any interventions. This finding suggests that the transmissibility of the virus might be high during the outbreak although the value of the transmissibility was not quantified. Therefore, in future control of dengue transmission, it calls for a high sensitivity and specificity of the surveillance systems including the diagnosis in hospitals or clinics, specimen collection and testing by PCR method or gene sequencing, and field epidemiological investigation. When an outbreak is confirmed and reported to the local CDC, our models could be employed to forecast the TAR and DO to simulate the future transmission scenarios and enable public health department to perform timely countermeasures.

### The effectiveness of interventions

Our study showed that the high transmissibility led to the rapid transmission of virus in the community in a short period although the density of the vector decreased after August. The high transmissibility also led to the control difficulty. Under this condition, LC would not be effective because there is a biological delay from larva to adult vector, and during that delay, the virus would have been transmitted widely among the population. But, to our knowledge, LC has a long term effectiveness. In the low or moderate transmissibility scenarios, LC would be valuable. In addition, high proportion of the asymptomatic infection leads to the disease transmission even though symptomatic individuals are totally isolated. Therefore, case isolation is not the primary intervention during an outbreak.

Fortunately, our study showed that the rHBR has distinctive effectiveness. However, because of the high transmissibility of the disease, the effectiveness is obvious when HBR is reduced to 17.2% of the initial value of the outbreak and would reach a satisfying effectiveness when reduced to 8.2%. In reality, HBR is hard to reduce down to 0% in short time. Although AVC is not the most effective among the single intervention, its TAR and DO were close to rHBR_100%_. Therefore, rHBR and LC are strongly recommended during the outbreak. On 18 August, 2018 (the outbreak reported date), the HBR was 12 per person per hour. But after intervention implemented, HBR was reduced to 4 per person per hour (down to 33%) and 1 per person per hour (down to 8.33%) in the following two days. These surveillance data are similar to the simulated data.

To our knowledge, when conducting AVC, the HBR will decrease. However, our outbreak did not provide a background for our investigation. During our outbreak control, AVC was conducted immediately covering the whole community, thus the procedure of interaction between AVC and HBR was shortened in a short time (a day). The results of our models also showed that the TAR and DO of rHBR (100%), AVC, and rHBR (100%)+AVC were almost the same (Table 3). Therefore, the interaction mechanism between AVC and rHBR remains unknown. More researches are needed to quantify the interaction.

### Limitations

There are several limitations in our study. Firstly, our simulation model has dynamics the dengue incidence using the ODE model based on a small scale outbreak in a community. The small population based modelling might lead to some uncertainty. Fortunately, our previously research found that the simulation results would be stable when the population is larger than 2000 people [24]. In addition, the transmission among the population was clear by the virological- and epidemiological-based evidence. We got gene sequences data of about 89% confirmed cases during the outbreak. The vector surveillance data during the outbreak and in the year of 2018 also provided a solid grounding in the foundations of model. These efforts have improved the reliability of our modelling and effectiveness assessment of intervention. Secondly, the effect of the Jongdari typhoon (23 Jul—4 Aug) was not taken into account, but this potentially may influence the number of dengue confirmed cases.

### Conclusions

Without intervention, DENV-1 could transmit rapidly within a short period of time and lead to high attack rate in community in China. The ODE models can be used to simulate the incidence dynamic of dengue outbreak and to assess the effectiveness of the countermeasures. AVC or rHBR should be recommended as primary interventions to control the dengue virus transmission rapidly at the early stage of an outbreak. Bed net or mosquito repellents were encouraged to be used in the community to reduce HBR, and space spraying of insecticides was recommended to control adult vector during the outbreak.

## Supporting information

S1 Checklist STROBE Statement—Checklist of items that should be included in reports of observational studies. (DOCX)

S1 Table The names and the GenBank accession numbers of the 25 viruses. (XLSX)

S1 Fig Curve fitting of data from the baseline of the outbreak simulation from August 11 to 19, 2018. (TIF)

S2 Fig Sensitivity analysis to 8 parameters based on the 1000 runs of the model. A, sensitivity analysis to parameter *ω*_*p*_; B, sensitivity analysis to parameter *ω*_*m*_; C, sensitivity analysis to parameter *q*; D, sensitivity analysis to parameter *γ*; E, sensitivity analysis to parameter *γ*’; F, sensitivity analysis to parameter *a*; G, sensitivity analysis to parameter *n*; H, sensitivity analysis to parameter *b*.
(TIF)

## References

[1] Guzman MG, Halstead SB, Artsob H, Buchy P, Farrar J, Gubler DJ, et al Dengue: a continuing global threat. Nature reviews Microbiology. 2010;8(12 Suppl):S7–16. Epub 2010/11/17. 10.1038/nrmicro2460 21079655 PMC4333201

[2] Lai S, Huang Z, Zhou H, Anders KL, Perkins TA, Yin W, et al The changing epidemiology of dengue in China, 1990–2014: a descriptive analysis of 25 years of nationwide surveillance data. BMC medicine. 2015;13:100 Epub 2015/05/01. 10.1186/s12916-015-0336-1 25925417 PMC4431043

[3] Bhatt S, Gething PW, Brady OJ, Messina JP, Farlow AW, Moyes CL, et al The global distribution and burden of dengue. Nature. 2013;496(7446):504–7. 10.1038/nature12060 23563266 PMC3651993

[4] Simmons CP, Farrar JJ, Nguyen vV, Wills B. Dengue. The New England journal of medicine. 2012;366(15):1423–32. 10.1056/NEJMra1110265 . 22494122

[5] Brady OJ, Gething PW, Bhatt S, Messina JP, Brownstein JS, Hoen AG, et al Refining the global spatial limits of dengue virus transmission by evidence-based consensus. PLoS neglected tropical diseases. 2012;6(8):e1760 10.1371/journal.pntd.0001760 22880140 PMC3413714

[6] Zhu G, Xiao J, Zhang B, Liu T, Lin H, Li X, et al The spatiotemporal transmission of dengue and its driving mechanism: A case study on the 2014 dengue outbreak in Guangdong, China. The Science of the total environment. 2018;622–623:252–9. 10.1016/j.scitotenv.2017.11.314 . 29216466

[7] Wu JY, Lun ZR, James AA, Chen XG. Dengue Fever in mainland China. The American journal of tropical medicine and hygiene. 2010;83(3):664–71. 10.4269/ajtmh.2010.09-0755 20810836 PMC2929067

[8] Esteva L, Vargas C. Analysis of a dengue disease transmission model. Mathematical biosciences. 1998;150(2):131–51. Epub 1998/07/10. . 9656647 10.1016/s0025-5564(98)10003-2

[9] Johansson MA, Hombach J, Cummings DA. Models of the impact of dengue vaccines: a review of current research and potential approaches. Vaccine. 2011;29(35):5860–8. 10.1016/j.vaccine.2011.06.042 21699949 PMC4327892

[10] Newton EA, Reiter P. A model of the transmission of dengue fever with an evaluation of the impact of ultra-low volume (ULV) insecticide applications on dengue epidemics. The American journal of tropical medicine and hygiene. 1992;47(6):709–20. Epub 1992/12/01. 10.4269/ajtmh.1992.47.709 . 1361721

[11] Cheng Q, Jing Q, Spear RC, Marshall JM, Yang Z, Gong P. Climate and the Timing of Imported Cases as Determinants of the Dengue Outbreak in Guangzhou, 2014: Evidence from a Mathematical Model. PLoS neglected tropical diseases. 2016;10(2):e0004417 10.1371/journal.pntd.0004417 26863623 PMC4749339

[12] Tang B, Xiao Y, Tang S, Wu J. Modelling weekly vector control against Dengue in the Guangdong Province of China. Journal of theoretical biology. 2016;410:65–76. 10.1016/j.jtbi.2016.09.012 . 27650706

[13] Andraud M, Hens N, Marais C, Beutels P. Dynamic epidemiological models for dengue transmission: a systematic review of structural approaches. PloS one. 2012;7(11):e49085 Epub 2012/11/10. 10.1371/journal.pone.0049085 23139836 PMC3490912

[14] Zhao J, Liu R, Chen S, Chen T. [A model for evaluation of key measures for control of chikungunya fever outbreak in China]. Zhonghua liu xing bing xue za zhi = Zhonghua liuxingbingxue zazhi. 2015;36(11):1253–7. Epub 2016/02/07. . 26850246

[15] Wang T, Wang M, Shu B, Chen XQ, Luo L, Wang JY, et al Evaluation of inapparent dengue infections during an outbreak in Southern China. PLoS neglected tropical diseases. 2015;9(3):e0003677 Epub 2015/04/01. 10.1371/journal.pntd.0003677 25826297 PMC4380470

[16] Bosio CF, Thomas RE, Grimstad PR, Rai KS. Variation in the efficiency of vertical transmission of dengue-1 virus by strains of Aedes albopictus (Diptera: Culicidae). Journal of medical entomology. 1992;29(6):985–9. Epub 1992/11/01. 1460640. 10.1093/jmedent/29.6.985 1460640

[17] Chen T, Ka-Kit Leung R, Liu R, Chen F, Zhang X, Zhao J, et al Risk of imported Ebola virus disease in China. Travel medicine and infectious disease. 2014;12(6 Pt A):650–8. 10.1016/j.tmaid.2014.10.015 . 25467086

[18] Chen T, Leung RK, Zhou Z, Liu R, Zhang X, Zhang L. Investigation of key interventions for shigellosis outbreak control in China. PloS one. 2014;9(4):e95006 10.1371/journal.pone.0095006 24736407 PMC3988114

[19] Chen T, Gu H, Leung RK, Liu R, Chen Q, Wu Y, et al Evidence-Based interventions of Norovirus outbreaks in China. BMC public health. 2016;16(1):1072 10.1186/s12889-016-3716-3 . 27729034 PMC5059926

[20] Chen T, Zhao B, Liu R, Zhang X, Xie Z, Chen S. Simulation of key interventions for seasonal influenza outbreak control at school in Changsha, China. The Journal of international medical research. 2018:300060518764268 10.1177/0300060518764268 . 29569977 PMC7113490

[21] Liu R, Leung RK, Chen T, Zhang X, Chen F, Chen S, et al The Effectiveness of Age-Specific Isolation Policies on Epidemics of Influenza A (H1N1) in a Large City in Central South China. PloS one. 2015;10(7):e0132588 10.1371/journal.pone.0132588 26161740 PMC4498797

[22] Xu G, Dong H, Shi N, Liu S, Zhou A, Cheng Z, et al An outbreak of dengue virus serotype 1 infection in Cixi, Ningbo, People's Republic of China, 2004, associated with a traveler from Thailand and high density of Aedes albopictus. The American journal of tropical medicine and hygiene. 2007;76(6):1182–8. Epub 2007/06/09. . 17556633

[23] Yang F, Guo GZ, Chen JQ, Ma HW, Liu T, Huang DN, et al Molecular identification of the first local dengue fever outbreak in Shenzhen city, China: a potential imported vertical transmission from Southeast Asia? Epidemiology and infection. 2014;142(2):225–33. Epub 2013/04/17. 10.1017/S0950268813000897 . 23587429 PMC9151106

[24] Chen T, Chen T, Liu R, Xu C, Wang D, Chen F, et al Transmissibility of the Influenza Virus during Influenza Outbreaks and Related Asymptomatic Infection in Mainland China, 2005–2013. PloS one. 2016;11(11):e0166180 10.1371/journal.pone.0166180 . 27880774 PMC5120824

