# Supplementary figures and images for: Incidence dynamics and investigation of key interventions in a dengue outbreak in Ningbo City, China
Source: PLoS Negl Trop Dis. 2019 Aug 15;13(8):e0007659. doi: 10.1371/journal.pntd.0007659 (PMC6711548; doi:10.1371/journal.pntd.0007659)

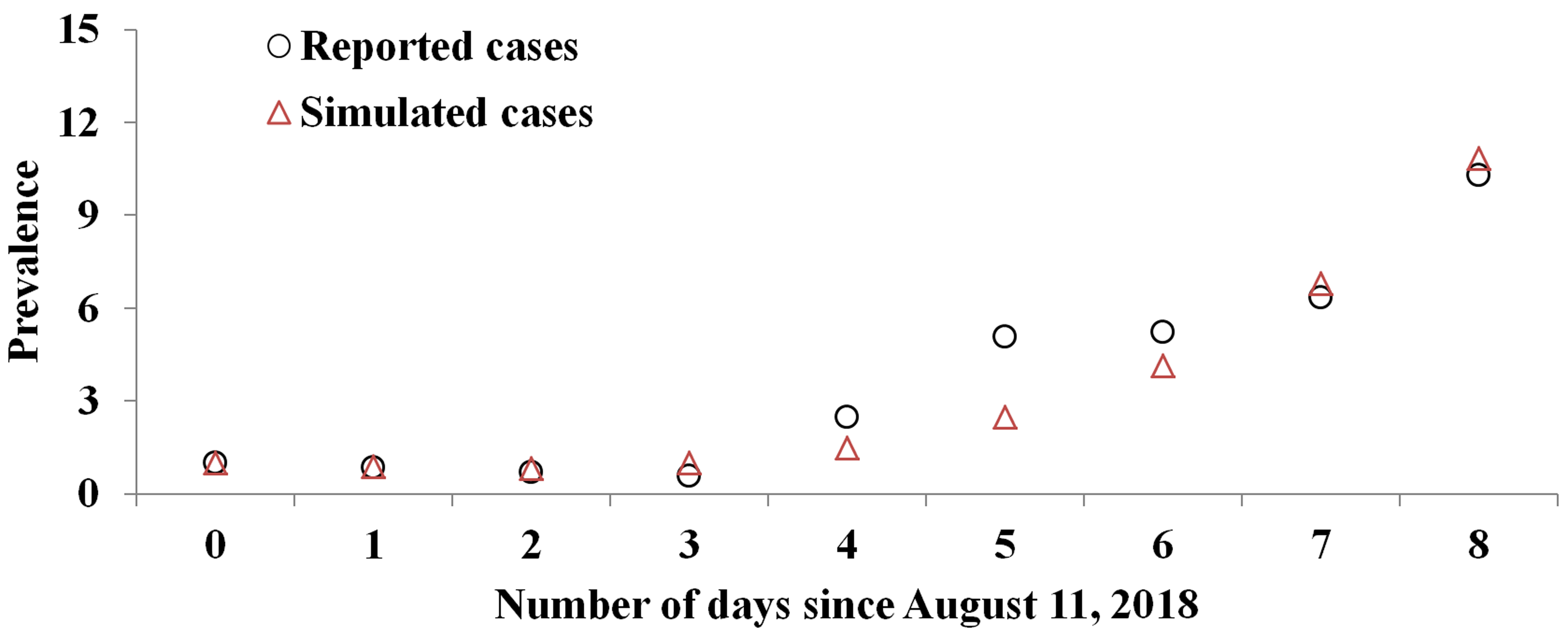

Supplement: S1 Fig — (TIF) [file pntd.0007659.s003.tif]

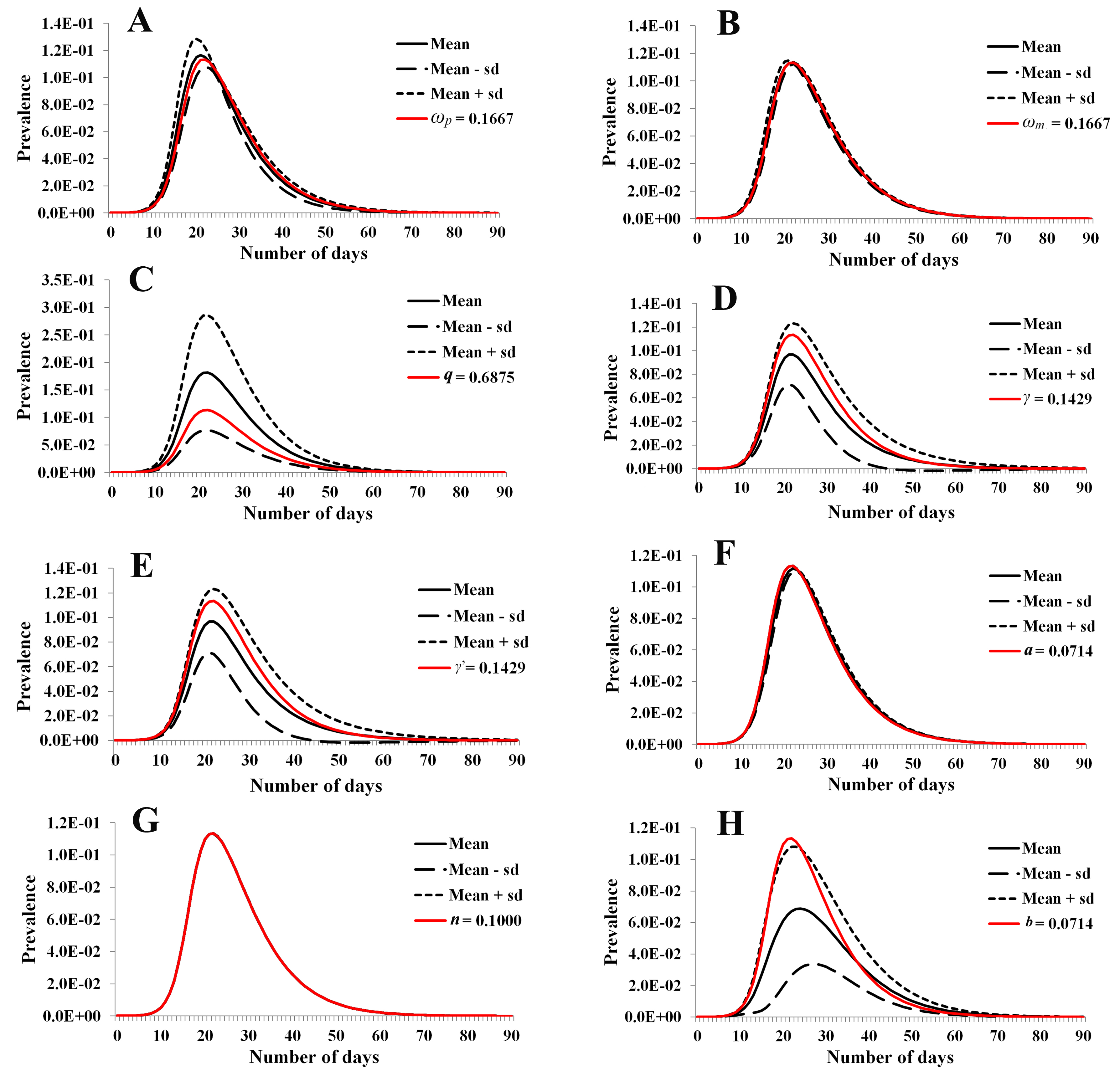

Supplement: S2 Fig — A, sensitivity analysis to parameter ωp; B, sensitivity analysis to parameter ωm; C, sensitivity analysis to parameter q; D, sensitivity analysis to parameter γ; E, sensitivity analysis to parameter γ’; F, sensitivity analysis to parameter a; G, sensitivity analysis to parameter n; H, sensitivity analysis to parameter b. (TIF) [file pntd.0007659.s004.tif]
